# Modulation of Angiogenic Processes by the Human Gammaherpesviruses, Epstein–Barr Virus and Kaposi’s Sarcoma-Associated Herpesvirus

**DOI:** 10.3389/fmicb.2019.01544

**Published:** 2019-07-12

**Authors:** Ricardo Rivera-Soto, Blossom Damania

**Affiliations:** ^1^Lineberger Comprehensive Cancer Center, The University of North Carolina at Chapel Hill, Chapel Hill, NC, United States; ^2^Curriculum in Genetics and Molecular Biology, The University of North Carolina at Chapel Hill, Chapel Hill, NC, United States; ^3^Department of Microbiology and Immunology, The University of North Carolina at Chapel Hill, Chapel Hill, NC, United States

**Keywords:** angiogenesis, Epstein–Barr virus, gammaherpesviruses, Kaposi’s sarcoma-associated herpesvirus, oncoviruses, vascular endothelial growth factor

## Abstract

Angiogenesis is the biological process by which new blood vessels are formed from pre-existing vessels. It is considered one of the classic hallmarks of cancer, as pathological angiogenesis provides oxygen and essential nutrients to growing tumors. Two of the seven known human oncoviruses, Epstein–Barr virus (EBV) and Kaposi’s sarcoma-associated herpesvirus (KSHV), belong to the *Gammaherpesvirinae* subfamily. Both viruses are associated with several malignancies including lymphomas, nasopharyngeal carcinomas, and Kaposi’s sarcoma. The viral genomes code for a plethora of viral factors, including proteins and non-coding RNAs, some of which have been shown to deregulate angiogenic pathways and promote tumor growth. In this review, we discuss the ability of both viruses to modulate the pro-angiogenic process.

## Introduction

The worldwide prevalence of human cancers caused by infectious agents has been estimated to be approximately 15–20% (Plummer et al., 2016). These infectious agents include the bacterium *Helicobacter pylori*, the parasite *Opisthorchis viverrini* and seven viruses. These human cancer-causing viruses, or oncoviruses, include both DNA and RNA viruses. Since the discovery of the first human oncovirus, Epstein–Barr virus (EBV), in 1964, six additional viruses have been found to be etiologically related to several malignancies (Mesri et al., 2014). The DNA oncoviruses are EBV, Kaposi’s sarcoma-associated herpesvirus (KSHV), human papillomaviruses (HPV), hepatitis B virus (HBV), and Merkel cell polyomavirus (MCV). The RNA oncoviruses are hepatitis C virus (HCV) and human T-lymphotropic virus (HTLV) type 1. Although significant improvements have been made with regards to prevention, diagnosis, and treatment of viral cancers, as evidenced by the vaccines against HBV and HPV, the multi-step process by which viruses can induce cell transformation and consequently cancer is not entirely understood. As illustrated by Mesri et al. (2014), viruses can hijack cellular processes to facilitate the dysregulated growth of cells leading to the formation of tumors. Also, to modulate cell proliferation and prevent cell death, oncoviruses promote angiogenesis, providing the tumors with a continuous supply of nutrients.

**FIGURE 1:**
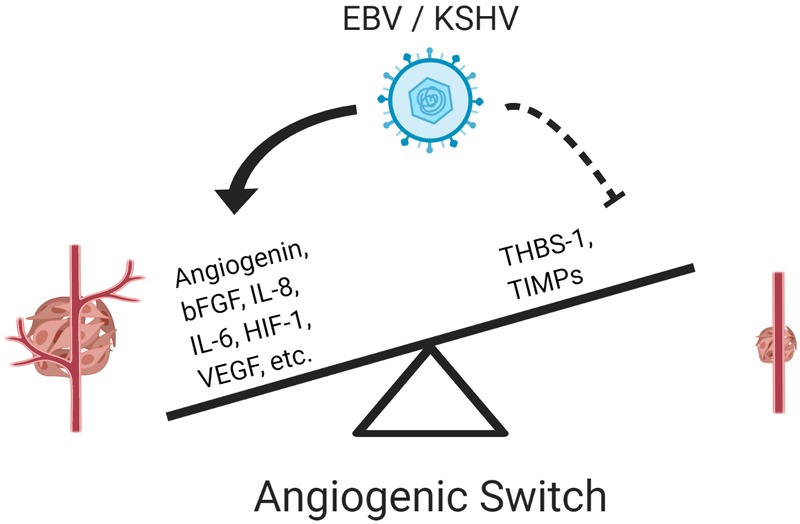
Epstein–Barr virus and KSHV induce an angiogenic switch. Both viruses activate pro-angiogenic factors and repress anti-angiogenic factors to activate the angiogenic switch promoting vessel formation and enhancing tumorigenesis.

Angiogenesis is a multi-step process that leads to the formation and growth of new blood vessels from pre-existing vessels. The angiogenic process is essential for physiological processes such as embryonic development, tissue repair during wound-healing, and the female reproductive cycle (Carmeliet and Jain, 2000). However, this process, which is tightly regulated by several pro- and anti-angiogenic factors, can be altered by cancer cells and result in enhanced tumorigenesis; the process itself is considered a hallmark of cancer (Hanahan and Weinberg, 2011). Angiogenesis occurs when the balance between pro- and anti-angiogenic factors is tilted toward the promotion of angiogenesis. This event is known as the “angiogenic switch,” and it is an essential step during the progression and metastasis of a malignant tumor, as it can provide oxygen and nutrients required for tumor growth (Bergers and Benjamin, 2003; Baeriswyl and Christofori, 2009; Bielenberg and Zetter, 2015). The activation of the angiogenic switch can occur in both viral and non-viral cancers, but in the former, oncoviral factors are to some extent responsible for tilting the balance toward the induction of angiogenesis (Figure 1).

The angiogenic process begins with the activation of endothelial cells. This process can be intercellularly initiated from the tumor cells by the transcription factors hypoxia-inducible factors (HIF) 1 and 2 (Krock et al., 2011). First, as a tumor grows, the tumor microenvironment becomes hypoxic allowing the stabilization of HIF-1α. During normoxic conditions, prolyl-hydroxylases (PHDs) hydrolyze HIF-1α, which can then be recognized by the von Hippel–Lindau (VHL) tumor suppressor. This leads to the ubiquitin-mediated degradation of HIF-1α. However, in the absence of oxygen (or during oncogenic activation), PHDs cannot hydrolyze HIF-1α, which result in HIF-1α translocating to the nucleus and dimerizing with HIF-1β to activate the HIF-responsive element (HRE)-containing promoters. Several pro- and anti-angiogenic factors including vascular endothelial growth factor (VEGF), fibroblast growth factors (FGF), platelet-derived growth factor (PDGF), and several interleukins (IL) such as IL-6 and IL-8 are induced by HIF-1 (Krock et al., 2011).

**FIGURE 2:**
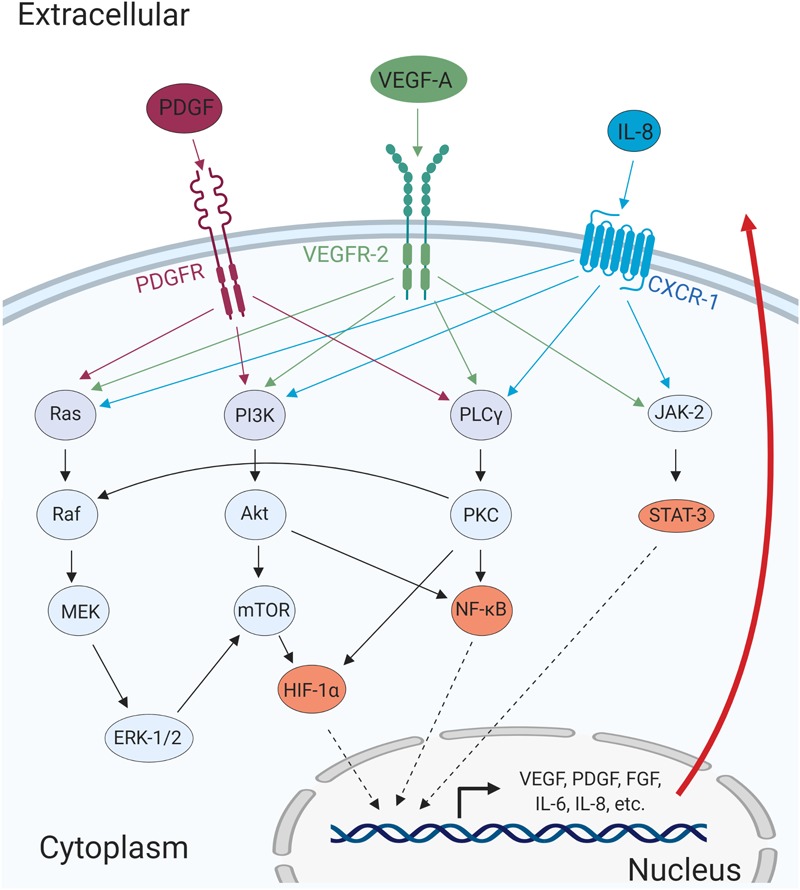
Major cellular pathways converge to promote angiogenic processes. The cellular receptors PDGFR, VEGFR-2, and CXCR-1, are activated upon ligand-binding and mediates activation of major signaling pathways such as MAPK/ERK, PI3K/Akt, PLCγ/PKC, and JAK/STAT. The activation of these pathways results in the transcription of pro-angiogenic genes that can promote cell migration, invasion, and angiogenesis in autocrine and paracrine manners.

Following the induction of expression, these pro-angiogenic factors are secreted from the tumor cells and intercellularly bind and activate their respective receptors in endothelial cells (Figure 2). Activation of the receptors such as VEGFR-2, FGFR, and Tie-2, stimulate the modulation of several signaling pathways including phosphatidylinositol 3-kinase (PI3K)/Akt, mitogen-activated protein kinase (MAPK)/extracellular-regulated kinase (ERK), and Janus kinase (JAK)/signal transducer and activator of transcription (STAT; Cross and Claesson-Welsh, 2001). This in turn further enhance angiogenesis by promoting cell survival, proliferation, and invasion (Cross and Claesson-Welsh, 2001).

In addition to the tumor cells, the endothelial sprouting cells forming the new vessels induce the expression and activation of pro-migratory factors such as matrix metalloproteinase enzymes (MMPs; described below). MMPs degrade extracellular matrix components and facilitate cell movement. The expansion and cell movement allow for the branching of blood vessels. Importantly, the secretion of pro-angiogenic factors by the tumor cells can also act as an attractant for the proliferating endothelial cells and recruitment of immune cells (Weis and Cheresh, 2011). Following the formation of the new blood vessel, pericytes are recruited and attached to newly matured endothelial cells (Weis and Cheresh, 2011).

However, due to the fast and uncontrolled growth, and continuous stimulation associated with pathological angiogenesis, these tumorigenic blood vessels acquire an aberrant morphology that includes excessive branching with defective basement membrane and uneven pericyte coverage (Azzi et al., 2013). These effects contribute to the tortuosity and leakiness of tumor-associated blood vessels facilitating metastasis (Azzi et al., 2013). Furthermore, the expansion of these new blood vessels allows for the formation of networks with existing vessels in a process known as anastomosis.

Two of the seven human oncoviruses, EBV and KSHV, belong to the subfamily of *Gammaherpesvirinae*. Both viruses have been identified as the etiologic agents of several malignancies that are dependent on the expression of pro-angiogenic factors. Though KSHV was discovered just 25 years ago, KSHV, as a potent inducer of angiogenesis, has been described to a greater extent than has EBV since Kaposi’s sarcoma, the cancer associated with KSHV, is one of the most angiogenic human tumors and is driven by KSHV-infected endothelial cells. In this review, we will describe the current knowledge of how both viruses, as a whole or via their proteins and non-coding RNAs, modulate angiogenic processes and how these might contribute to virus-induced tumorigenesis.

## Epstein–Barr Virus

Epstein–Barr virus (EBV) is a ubiquitous γ-1 herpesvirus that infects approximately 95% of the world’s population. EBV is associated with several malignancies that include B-, T-, and Natural Killer-cell lymphomas, post-transplant lymphoproliferative disease (PTLD), endemic Burkitt’s lymphoma, a subset of Hodgkin’s lymphomas, and the epithelial malignancies nasopharyngeal carcinoma (NPC) and gastric carcinoma (Young et al., 2016). Similar to other herpesviruses, EBV infection induces the formation of a viral episome that is tethered to the host genome and facilitates the establishment of a latent lifecycle.

Epstein–Barr virus induces different latency programs in infected cells. For example, *in vitro*, EBV infection transforms resting B-cells into immortalized lymphoblastoid cell lines (LCLs) that expresses several viral genes including all six Epstein–Barr nuclear antigens (EBNAs), latent membrane protein (LMP) 1, LMP2A and 2B, and EBV-encoded RNAs (EBER) 1 and 2. This type of latency, known as latency III, promotes the proliferation of the infected B-cells, allowing the replication of not only the host genome but also the viral episome. *In vivo*, latency III is associated with PTLD and diffuse large B-cell lymphomas (Young et al., 2016). This EBV-mediated proliferation mimics B-cell development in which cells enter the germinal center, but it is highly immunogenic. Instead, a less immunogenic latency program (latency II), as seen in the EBV-associated classical Hodgkin’s lymphoma, consists in the expression of EBNA1, LMP1, LMP2A and 2B, and EBER1–2. Importantly, this type of latency is also seen in the EBV-induced epithelial cancers, NPC, and gastric carcinomas.

Memory B-cells are thought to be the reservoir for EBV, and in these cells, the virus induces two distinct programs, latency 0 and latency I. In latency 0, which is specific to resting B-cells, only EBER1–2 are expressed. On the other hand, a dividing memory B-cell experiences latency I, in which EBER1–2 and EBNA1 are expressed. The expression of EBNA1 is sufficient for the replication and maintenance of the viral episome. Burkitt’s lymphoma shows a latency-I expression profile. A specific latency pattern is not needed for EBV to induce cell transformation since the malignant cells show different gene expression profiles (Young et al., 2016).

Several groups have explored the effect of EBV infection in inducing angiogenesis. For example, infection of epithelial cells, the cells of origin for EBV-induced gastric and nasopharyngeal carcinomas, induces the formation of vascular mimicry that correlates with tumor growth (Xiang et al., 2018). Vascular mimicry is the process by which non-endothelial cells form tubular networks and allow for blood flow independent of endothelial cells (Dunleavey and Dudley, 2012). Furthermore, EBV-positive gastric carcinomas show an aberrant regulation of epigenetic markers of enhancers associated with angiogenic processes; these malignancies also display silencing of tumors suppressors and anti-angiogenic factors such as phosphatase and tensin homolog (PTEN) and thrombospondin (THBS) 1 (Kang et al., 2002; Okabe et al., 2017).

Further evidence has demonstrated that EBV-positive NPC cells are more tumorigenic and angiogenic *in vivo* than EBV-negative NPC cells (Ye et al., 2018). EBV is associated with high levels of VEGF and reduced survival of Hodgkin’s lymphoma patients (Koh et al., 2018). Angiogenic processes are also involved in the development of B-cell lymphomas in mice caused by the engraftment of EBV-transformed LCLs (Woodford et al., 2004). These studies outline a role for EBV in inducing pro-angiogenic processes that enhance tumorigenesis. Elucidating the process by which the individual viral proteins and non-coding RNAs modulate angiogenesis is essential for developing effective therapies. We describe a few of these viral factors below.

### Latent Membrane Protein 1

**FIGURE 3:**
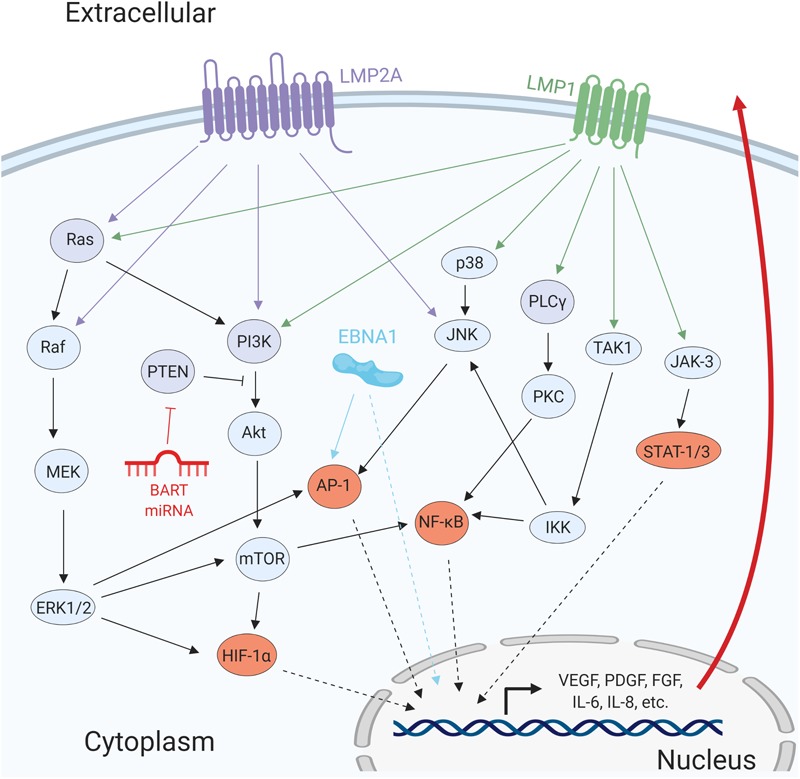
Epstein–Barr virus proteins and miRNAs promote angiogenesis by activating several cellular signaling pathways. The viral transmembrane proteins LMP1 and LMP2A promote the activation of several signaling pathways including MAPK/ERK, PI3K/Akt, PLCγ/PKC, and JAK/STAT. EBNA1 directly promotes activation of pro-angiogenic genes and transcription factors such as AP-1. The BART miRNA targets the negative regulator of PI3K/Akt, PTEN. Activation of these signaling pathways results in the promotion of pro-survival and pro-angiogenic factors.

A major oncogenic protein of EBV is LMP1. LMP1 has transforming properties in cultured cell lines, and it is expressed in most EBV-associated cancers (Wang et al., 1985). LMP1, a mimic of cellular CD40, is a constitutively active member of the tumor necrosis factor receptor (TNFR) family with the ability to activate signaling pathways including NF-κB, PI3K/Akt, MAPK/ERK, JAK/STAT, and c-Jun N-terminal kinase (JNK) (Figure 3; Mosialos et al., 1995; Devergne et al., 1996; Gires et al., 1999; Dawson et al., 2003; Mainou et al., 2005). Activation of these pathways has multiple effects in the infected cells, including the modulation of apoptosis, cell migration, cell cycle progression, and angiogenesis (Table 1). One line of evidence that LMP1 is involved in inducing angiogenesis can be demonstrated by introducing LMP1 into LMP1-negative NPC cells. The co-culture of LMP1-expressing NPC and human umbilical vein endothelial cells (HUVECs) induces the formation of endothelial cell tubular structures on Matrigel, demonstrating the contribution of the viral protein to the angiogenic process (Yang et al., 2015).

**Table 1:** Major pro- and anti-angiogenic factors modulated by EBV viral factors.

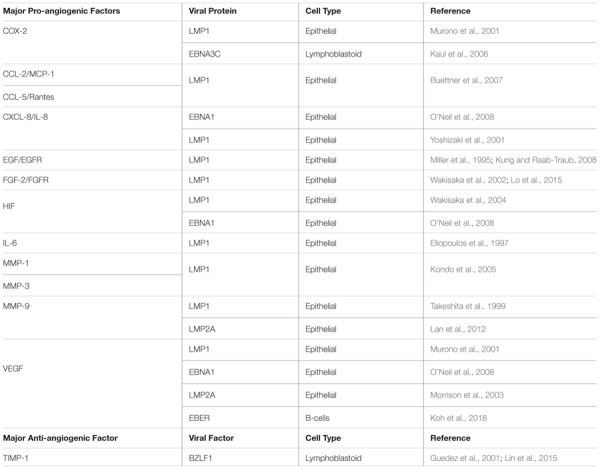

In NPC tumor tissues, VEGF is highly expressed, and it correlates with microvessel density (Wakisaka et al., 1999; Guang-Wu et al., 2000; Qian et al., 2000). Moreover, through the activation of VEGF/VEGFR-1, LMP1 induces vascular mimicry, which is associated with poorer prognosis (Xu et al., 2018). Additionally, in diffuse large B-cell lymphoma patients, EBV infection, and VEGF-A expression associates with aggressive subtypes and shorter survival times (Paydas et al., 2008). As mentioned in the introduction, induction of HIF proteins and VEGF is one of the essential steps for the promotion of angiogenesis; thus, it is not surprising that several signaling pathways converge to induce the expression and stabilization of these proteins (Carmeliet, 2005; Krock et al., 2011). In nasopharyngeal epithelial cells, LMP1 induces the expression of HIF-1α and VEGF by activating MAPK/ERK, JNKs and JAK/STAT signaling pathways (Gires et al., 1999; Wakisaka et al., 2004; Wang et al., 2010; Yang et al., 2015). Thus, LMP1 activates multiple signaling pathways to induce VEGF, which is usually associated with more aggressive disease.

Furthermore, LMP1 induces the production of reactive oxygen species (ROS) such as H_2_O_2_, which mediates the stabilization and translocation of HIF-1α (Chandel et al., 2000; Wakisaka et al., 2004). Moreover, LMP1 induces the activation of HIF-1α and PI3K/Akt through the expression of the chemokine (C-C motif) ligand (CCL) 5 (also known as RANTES; Buettner et al., 2007; Ma et al., 2018). Also, LMP1 can upregulate the levels of the E3 ubiquitin ligase Siah-1 (Kondo et al., 2006), which targets the HIF-1α negative regulators’ PHDs 1 and 3 for degradation (Nakayama et al., 2004). Therefore, LMP1 increases the levels of HIF-1α not only by promoting its expression but also by downregulating the repressors involved in the control of HIF-1α.

For proper induction of angiogenesis, LMP1 requires the activation of NF-κB. The activation of NF-κB is mediated by LMP1’s C-terminal-activating regions, CTAR1 and CTAR2, which can constitutively associate with tumor necrosis factor receptor-associated factors (TRAFs). This interaction leads to the activation of the NF-κB signaling cascade, which promotes the expression of genes with multiple functions, including induction of angiogenesis. One angiogenic factor induced by LMP1 that requires NF-κB is cyclooxygenase-2 (COX-2). Activation of COX-2 in LMP1-expressing NPC cells leads to an increase in the production of the angiogenic factors prostaglandin E2 (PGE2) and VEGF (Murono et al., 2001). Furthermore, in NPC tissues, the expression of COX-2 correlates with LMP1, whereas LMP1-negative NPC infrequently expresses COX-2 (Murono et al., 2001; Fendri et al., 2008).

Similar to COX-2, the expression of IL-8 is, to some extent, dependent on NF-κB as the NF-κB-binding sites in the IL-8 promoter are necessary for LMP1-induced expression (Yoshizaki et al., 2001). Interleukin-8 (also known as CXCL-8) is a proinflammatory chemokine that has the pro-angiogenic activity of activating CXCR-1 (Figure 2; Koch et al., 1992; Waugh and Wilson, 2008; Shi and Wei, 2016). Importantly, the expression of LMP1 also correlates with IL-8 in NPC tumor tissues, and it is associated with the formation of microvessels and poor prognosis in NPC patients (Xie et al., 2010).

Furthermore, LMP1 induces the expression of the pro-angiogenic factors FGF-2 and epidermal growth factor (EGF) receptor (EGFR) (Miller et al., 1995; Wakisaka et al., 2002; Thornburg and Raab-Traub, 2007; Kung and Raab-Traub, 2008; Lo et al., 2015). This induction is also mediated through the activation of NF-κB, and the activation of FGF-2/FGFR-1 signaling is important for the transformation of NPC cells (Lo et al., 2015). FGF-2, which can also be present in LMP1-induced exosomes, can act in both paracrine and autocrine manners to induce the expression and secretion of VEGF, promoting the activation of endothelial cells (Seghezzi et al., 1998; Ceccarelli et al., 2007). In addition to NF-κB, STAT-3, and protein kinase C gamma (PKCγ) are essential for the LMP1-CTAR1-induced augmentation of EGFR activation (Kung and Raab-Traub, 2008). It is possible that through the activation of EGFR in NPC cells, EBV enhances calcium signaling that promotes VEGF expression (Ye et al., 2018). Together, these studies suggest that induction of both FGFR and EGFR contributes to LMP1-mediated angiogenesis.

An additional pro-angiogenic growth factor that LMP1 induces in epithelial cells is the hepatocyte growth factor receptor (HGFR, also known as c-Met). The LMP1-induced expression of c-Met is mediated through activation of the Ets-1 transcription factor (Horikawa et al., 2001). The expression of c-Met, LMP1, and Ets-1 are correlated in NPC tumor tissues. c-Met plays an important role in both angiogenesis and metastasis, and it is usually associated with poor prognosis (Ding et al., 2003; You and McDonald, 2008). Activation of c-Met in endothelial cells can lead to an increase of VEGF and suppression of the anti-angiogenic protein THBS-1, thus enhancing the angiogenic process (Zhang et al., 2003). In EBV-positive gastric carcinomas, the overexpression of c-Met may contribute to angiogenesis. However, the EBV protein that induces c-Met may not be LMP1, as it is rarely expressed in these cells (Kijima et al., 2002; Luo et al., 2006). Thus, EBV, through LMP1 and other viral genes, induces several growth factor receptors that contribute to the promotion of angiogenesis.

Matrix metalloproteinase enzymes (MMPs) are a group of zinc-containing endopeptidases capable of degrading ECM components and mediating pericyte detachment from vessels undergoing angiogenesis (Rundhaug, 2005). This group of proteins is essential for angiogenesis, but they also play a critical role in metastasis by cleaving the ectodomain of vascular endothelial (VE)-cadherin that maintains cell-cell adhesions (Shiomi and Okada, 2003). The expression of MMP-9 is induced by the CTAR1–2 domains of LMP1 (Yoshizaki et al., 1998; Takeshita et al., 1999). Importantly, the NF-κB and AP-1 binding sites of the MMP-9 promoter are necessary for MMP-9 expression, suggesting the involvement of both pathways. In addition to MMP-9, MMP-1 is also highly expressed in NPC and modulated by LMP1 (Lu et al., 2003; Kondo et al., 2005). Interestingly, a polymorphism in the MMP-1 promoter of NPC cells creates an Ets binding site, and its high expression is associated with poor prognosis (Kondo et al., 2005). In addition to increasing MMP-1 levels directly, LMP1 can also increase the production of MMP-3, which cleaves and consequently activates MMP-1. Expression of both MMP-1 and MMP-3 is dependent on AP-1, and Ets transcription factors, as mutations to their binding sites or co-expression of double-negative mutants perturb their expression (Kondo et al., 2005).

LMP1 also induces the expression of other pro-migratory factors. Among these, is Twist, which increases cell migration and drives the epithelial-mesenchymal transition (Horikawa et al., 2007). Clinical samples show a high correlation between Twist and LMP1 expression in metastatic NPC (Horikawa et al., 2007). In addition to being a master regulator in embryogenesis (a process that requires extensive pro-angiogenic activity), Twist has been shown to mediate angiogenesis in an *in vivo* model of breast cancer (Mironchik et al., 2005). In NPC tissues, LMP1 induces the receptor for advanced glycation end products (RAGE; Tsuji et al., 2008). High microvessel counts, as determined by von Willebrand factor (vWF) staining, are associated with high levels of RAGE, LMP1, and lymph node metastasis, suggesting a role for RAGE in EBV-induced angiogenesis (Yamagishi et al., 1997; Tsuji et al., 2008). Furthermore, LMP1 promotes the expression of the soluble TNFR Decoy receptor (Dcr) 3, which in endothelial cells promotes angiogenesis by inhibiting TNF-like molecule 1A (TL1A; Ho et al., 2009). The expression of Dcr3 in NPC cells is associated with increased cell migration and invasion (Ho et al., 2009). In order for proper angiogenesis to occur, cells must migrate and invade through the extracellular matrix, and LMP1 induces the expression of several factors that facilitate this process.

Tumor viruses such as EBV can induce the secretion of exosomes that facilitate intercellular communication (Keryer-Bibens et al., 2006; Meckes et al., 2010). Exosomes are 40- to 100-nm secreted endosomal vesicles containing proteins, mRNAs, and microRNAs that can influence the tumor microenvironment (Schorey and Bhatnagar, 2008). Exosomes released from LMP1-positive NPC cells contain LMP1, cellular miRNAs, EBERs, signal transduction proteins and HIF-1α (Ceccarelli et al., 2007; Meckes et al., 2010; Aga et al., 2014; Yoon et al., 2016). The enclosed proteins maintain their activity and can increase the pathogenicity of the recipient cells (e.g., endothelial cells) by promoting processes such as migration, invasion, and angiogenesis (Meckes et al., 2010). Retinal pigmental epithelial cells grown in the presence of exosomes from the EBV-positive B-cell line Raji have higher levels of VEGF which was due in part to the cellular miR-155 contained in those vesicles (Yoon et al., 2016). It has been demonstrated that LMP1 induces expression of miR-155 by activating NF-κB signaling (Gatto et al., 2008; Lu et al., 2008). Secretion of exosomes provides a mechanism by which viral cancers can influence the conditions of the tumor microenvironment without the need for viral propagation.

Altogether, these studies support a model in which LMP1 promotes the activation of an arsenal of factors that contribute to EBV-induced angiogenesis.

### Latent Membrane Protein 2A

The transmembrane protein LMP2A is expressed during latency II and III and induces ligand-independent activation of the Src and Syk family of proteins by mimicking an active B-cell receptor (Portis and Longnecker, 2004). This interaction mediates the activation of Ras, PI3K, and JNK (Figure 3). Consequently, expression of LMP2A in murine B-cells drives the expression of genes promoting cell proliferation and survival that are common in EBV-positive Hodgkin’s lymphoma cells (Portis et al., 2003). These observations suggest that LMP2A may play a significant role in Hodgkin’s lymphoma development. Similarly, epithelial cells expressing LMP2A have higher PI3K/Akt and Wnt signaling activity, which can result in the expression of VEGF (Morrison et al., 2003). Notch activation, which has been shown to have pro-angiogenic effects, can be promoted by LMP2A-mediated induction of Notch ligand Delta and transcription factor Hes-1 (Sakakibara and Tosato, 2009). In addition to LMP1, LMP2A also contributes to vascular mimicry of EBV-infected epithelial cells. However, in contrast to LMP1, LMP2A-mediated induction of vascular mimicry is independent of VEGF but relies on HIF-1α through the activation of PI3K/Akt signaling (Xiang et al., 2018). Additionally, LMP2A promotes the invasiveness of NPC cells through the ERK-dependent expression of MMP-9 (Lan et al., 2012). Thus, LMP2A contributes to EBV-induced angiogenesis and may be an essential player for lymphomagenesis.

### EBV Nuclear Antigen 1

The EBV nuclear antigen (EBNA) 1 is an essential protein necessary for viral maintenance and replication (reviewed in Wilson et al., 2018). It plays the essential role of tethering the viral episome to the host genome and acting as a modulator of expression of viral and cellular genes (Wood et al., 2007; Frappier, 2015). Through the modulation of gene expression, EBNA1 enhances STAT-1 activation and interferes with transforming growth factor (TGF)-β signaling as TGF-β1-responsive genes are repressed in EBNA1-expressing cells (Wood et al., 2007). Furthermore, EBNA1 promotes the activation of the AP-1 transcription factor in epithelial cells, resulting in the expression of its pro-angiogenic target genes including IL-8, VEGF, and HIF-1α (O’Neil et al., 2008). The expression and secretion of IL-8 and VEGF from EBNA-1 expressing epithelial cells induces angiogenic phenotypes in an intercellular manner (O’Neil et al., 2008). Thus, in addition to being essential for viral maintenance, EBNA1 is also involved in the modulation of the pro-angiogenic process.

### EBV Nuclear Antigen 3

EBNA3 contains three spliced variants (A, B, and C) that are encoded in tandem and expressed during latency III. These variants are a family of transcription co-regulators that do not bind directly to DNA but instead use other proteins to modulate transcription. For example, EBNA3C interacts with the nucleoside diphosphate kinase (Nm23-H1) to induce the expression of the pro-angiogenic factor COX-2 in an NF-κB-dependent manner (Kaul et al., 2006). Importantly, expression of EBNA3C alone does not increase the levels of COX-2, demonstrating the requirement for an additional cofactor. EBNA3C also modulates JAK/STAT signaling by upregulating STAT-3 expression and downregulating the negative regulator of STAT-3, protein inhibitor of activated STAT 1 (PIAS-1; Zhao et al., 2011). Although not much is known about EBNA3 and angiogenesis, these studies have demonstrated a possible role of the viral protein in contributing to EBV-induced tumorigenesis.

### EBV Non-coding RNAs

The EBV-encoded small RNAs (EBERs) 1 and 2 are highly abundant viral transcripts in latently infected cells that are used for the detection of EBV-infected tissues (reviewed in Iwakiri and Takada, 2010). Although they are not essential for the *in vitro* transformation of B-cells, several studies have shown that they are involved in preventing apoptosis while promoting cell proliferation, tumor growth, and possible angiogenesis (Swaminathan et al., 1991; Laing et al., 2002; Nanbo et al., 2002; Iwakiri et al., 2003). With regards to angiogenesis, EBER expression has been directly correlated to VEGF expression in Hodgkin’s lymphoma patients (Koh et al., 2018). Importantly, EBER-containing exosomes secreted from NPC cells can be transferred to surrounding endothelial cells and singularly induce the expression of vascular cell adhesion molecule (VCAM) 1, which promotes angiogenic processes in *in vitro* and *in vivo* models (Cheng et al., 2019).

Epstein–Barr virus encodes at least 48 mature miRNAs from two different regions of the viral genome. Four are produced from the *BamHI* fragment H rightward reading frame (BHRF) 1 gene and the rest from *BamHI* fragment A rightward transcript (BART; Chen et al., 2010). EBV miRNAs can target both cellular and viral mRNAs to modulate the host immune response and maintain viral latency (reviewed in Wang et al., 2018). Additionally, several lines of evidence suggest that miRNAs are involved in promoting survival and tumorigenesis. In NPC cells, the miR BART1 targets both AMP-activated protein kinase (AMPK) α1 and PTEN, which results in the activation of downstream targets including mammalian target of rapamycin (mTOR) and MAPK/ERK (Cai et al., 2015; Lyu et al., 2018). Activation of these pathways promotes epithelial to mesenchymal transition and enhances glycolysis, cell migration, invasion, and angiogenesis. Altogether, these studies demonstrate that both EBERs and EBV miRNAs might contribute to EBV-induced angiogenesis by acting in a paracrine manner or by suppressing tumor suppressors.

### BZLF1 and BRLF1

The BZLF1 (Zta) and BRLF1 (Rta) viral transcription factors are two lytic proteins essential for the induction of the lytic cycle. Although both genes are dispensable for immortalization of primary B-cells *in vitro*, they are important for promoting tumor growth of LCLs in severe combined immunodeficient mice (Hong et al., 2005a). In contrast to LCLs infected with WT EBV, cells infected with BZLF1- or BRLF1-deleted virus have lower levels of VEGF secretion, and supernatant from these cells has reduced ability to promote tubule formation in endothelial cells (Hong et al., 2005b). Even though most EBV-associated tumor cells have the virus in latent form, it is not uncommon to find a small subset of lytically infected cells. Several lines of evidence have suggested that this small percentage of cells is necessary for proper tumor formation, as they contribute in a paracrine manner by secreting growth factors (Ma et al., 2011; McHugh et al., 2017; Cohen et al., 2018).

In addition to promoting VEGF, BZLF1 induces the expression of tissue inhibitor of metalloproteinase (TIMP) 1, which is involved in the modulation of MMPs but also plays a role in viral tumorigenesis by acting as an anti-apoptotic protein (Guedez et al., 2001; Lin et al., 2015). Thus, these two lytic proteins may contribute to EBV-induced tumorigenesis by modulating the expression of factors involved in angiogenesis.

## Kaposi’s Sarcoma-Associated Herpesvirus

Kaposi’s sarcoma-associated herpesvirus is a γ-2 herpesvirus associated with three human malignancies: the endothelial cell-driven cancer Kaposi’s sarcoma (KS) and two B-cell lymphoproliferative diseases: Multicentric Castleman’s disease (MCD) and primary effusion lymphoma (PEL). Like EBV, KSHV infection is lifelong, and diseases mostly arise in the context of immunosuppression (Dittmer and Damania, 2016). Following infection, the viruses establish a latent cycle expressing only a handful of genes. The latency locus includes open reading frame (ORF)71, ORF72, ORF73, ORF K12, and miRNAs. Upon induction of lytic replication, which is controlled by the protein product of ORF50, transcription of the viral genome occurs in an orderly fashion (Dittmer and Damania, 2016). As discussed with regard to EBV, although KSHV-associated tumor cells are latently infected, a subset of them undergo lytic replication, which is hypothesized to contribute to viral tumorigenesis by providing growth factors in a paracrine manner (Mesri et al., 2014). Many of these pro-tumorigenic factors are directly linked to the induction of angiogenic processes (Dimaio and Lagunoff, 2012; Purushothaman et al., 2016).

Initial studies in KS and PEL illustrated the importance of tumor cells in inducing VEGF and VEGFR (Brown et al., 1996; Cornali et al., 1996; Flore et al., 1998; Aoki and Tosato, 1999; Masood et al., 2002). VEGF can enhance the infectivity of the virus, and its expression is increased as early as 30 min post-infection, suggesting a role in mediating infection (Sivakumar et al., 2008). Following infection, KSHV triggers the reactivation of silenced genes such as PAX2. Generally restricted to embryogenesis, KSHV-mediated induction of PAX2 leads to the expression of CCL-2 and Akt, which contributes to the angiogenic and invasiveness potential of endothelial cells (Fonsato et al., 2008). Furthermore, KSHV-infected endothelial cells have increased PI3K/Akt activity, which is a major contributor to viral-induced tubule formation (Wang and Damania, 2008). Additionally, KSHV-infected endothelial cells express and secrete many other pro-angiogenic factors. Some of these proteins include HIF-1α, HIF-2α, IL-8, GRO-α, CCL-2, and angiopoietin-2 (Table 2; Lane et al., 2002; Carroll et al., 2006; Caselli et al., 2007, 2012; Ye et al., 2007; Fonsato et al., 2008).

**Table 2:** Major pro- and anti-angiogenic factors modulated by KSHV viral factors.

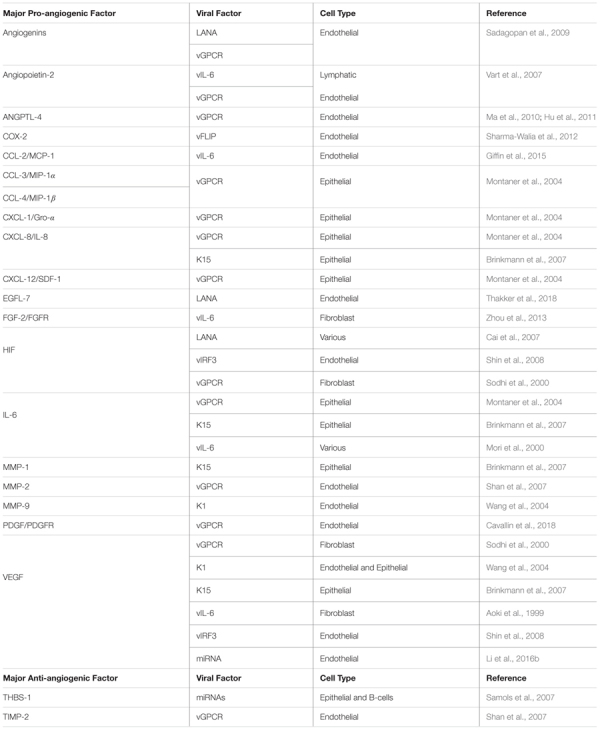

The demand for oxygen and nutrients during angiogenesis triggers ROS, which in turn contributes to the pathological process (Kim and Byzova, 2014). Consequently, KSHV tumor cells rely on the production of ROS, as treatment with antioxidants represses pro-angiogenic factors and decreases the formation of KS-like tumors in mice (Ma et al., 2009, 2013). For example, in an NF-κB-dependent but HIF-1α-independent manner, ROS and hypoxic conditions induce the expression of galectin-1, driving angiogenic phenotypes in KS-like tumors (Croci et al., 2012). Moreover, the redox functions of apurinic/apyrimidinic endonuclease (APE) 1, which can affect several types of transcription factors, are involved in KSHV-induced angiogenesis (Zhong et al., 2017). Thus, the production of ROS by KSHV-infected endothelial cells is needed for the formation of KS-like tumors and their vasculature.

Kaposi’s sarcoma-associated herpesvirus infection of endothelial cells promotes the expression of integrin subunits β1 and β3 (Dyson et al., 2007; DiMaio et al., 2011). In endothelial cells, integrins (e.g., αVβ1 and αVβ3) play a role in KSHV entry by binding to glycoprotein B (Kumar and Chandran, 2016). Activation of integrins such as αVβ3 induces phosphorylation of focal adhesion kinase (FAK) and tyrosine kinase Src, promoting the release of stored angiogenin (Ang) 2 (Ye et al., 2013). Ang-2 plays an essential role in the vasculature of KSHV tumors by serving as a ligand for the tyrosine kinase receptor Tie-2 (Yu et al., 2016). Activation of Tie-2 enhances VEGF signaling and induces activation of PI3K/Akt and MAPK/ERK pathways (Thurston and Daly, 2012). Thus, in endothelial cells, integrins facilitate viral binding and entry, and they are also involved in cell migration and angiogenesis.

**FIGURE 4:**
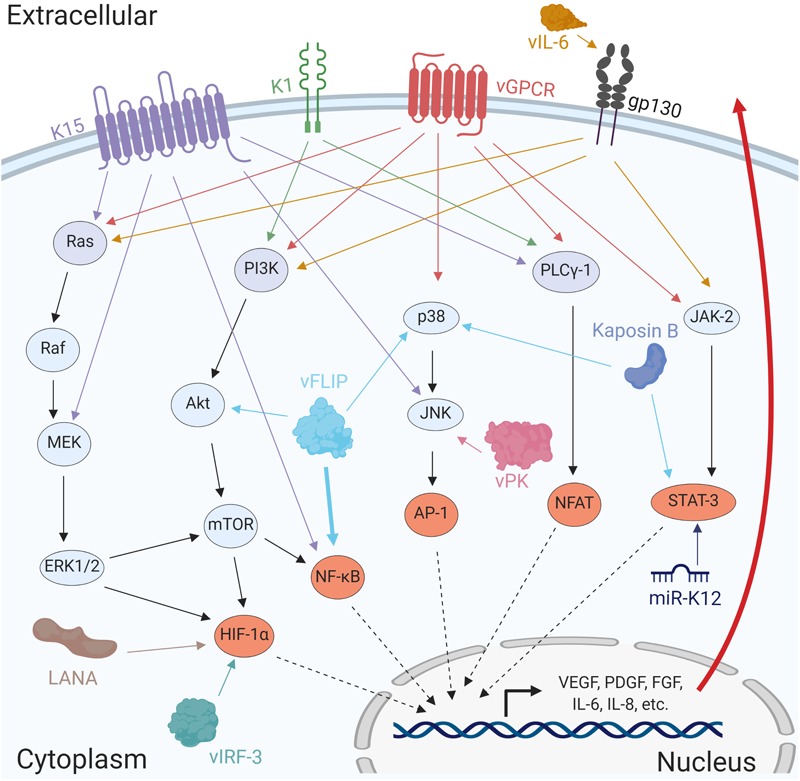
Several KSHV proteins and miRNAs promote angiogenesis by activating cellular signaling pathways. The viral factors LANA, vFLIP, Kaposin B, vIRF-3, and the miRNAs miR-K12 induces activation of major transcription factors such as HIF-1α, NF-κB, AP-1, and STAT-3. Additionally, the viral proteins K1, K15, vGPCR, vIL-6, and vPK cooperate in the activation of multiple signaling pathways promoting expression of angiogenic factors resulting in stronger viral tumorigenesis.

Furthermore, KSHV infection of endothelial cells promotes cell migration through the induction of MMP-1, MMP-2, and MMP-9 (Qian et al., 2007). This process is facilitated in part by the KSHV-induced activation of the AP-1 transcription factor. The disruption of cell-cell junctions enables cell migration, and in endothelial cells promotes vascular permeability. KSHV induces this process by modulating the expression or phosphorylation of VE-cadherin (Samaniego et al., 1998; Qian et al., 2008; Guilluy et al., 2011). Importantly, the viral proteins K1, K5, and vGPCR are significant contributors to these processes during both latent and lytic phases (Figure 4; Mansouri et al., 2008; Dwyer et al., 2011; Guilluy et al., 2011).

The ability of KSHV to infect a variety of cells, including endothelial and B-cells, is the starting point for a strong induction of angiogenic events facilitating tumor growth. As endothelial cells are the major components of blood vessels, manipulation and transformation of these cells is predicted to enhance viral tumorigenicity by, in part, promoting angiogenesis. Endothelial cells express several KSHV entry receptors including, integrins, xCT, and the tyrosine kinase receptor EphrinA2 (EphA2; reviewed in Kumar and Chandran, 2016). Viral binding to these receptors leads to the formation of signaling complexes promoting the interaction with PI3K, FAK, Src, and c-Cbl, thereby activating these signaling pathways (Chakraborty et al., 2012; Dutta et al., 2013). This process does not only allow for viral entry, but it is also predicted to modulate pro-angiogenic signaling as EphA2 is known to crosstalk with VEGF signaling (Barquilla and Pasquale, 2015).

In just a couple of decades, researchers have identified several viral factors, expressed during both latent and lytic cycles, that are involved in modulating angiogenesis and may represent viable targets against KSHV-associated malignancies. Next, we will review the current knowledge of how individual genes contribute to the angiogenic process.

### Latency-Associated Nuclear Antigen

The latency-associated nuclear antigen (LANA), encoded by ORF73 and homologous to EBNA1, is a major latency protein that tethers the KSHV episome to the host genome, allowing viral replication during regular cellular division (reviewed in Weidner-Glunde et al., 2017). LANA utilizes several mechanisms to promote angiogenesis, including inhibiting tumor suppressors and miRNAs to promote activation of transcription factors that modulate pro-angiogenic proteins.

The LANA-mediated inhibition of the VHL and p53 tumor suppressors promotes the stabilization of HIF-1α (Cai et al., 2006, 2007). LANA also stabilizes the Notch effector Hey-1, preventing its degradation, and facilitating Notch signaling (Wang et al., 2014). Furthermore, LANA sequesters death-associated protein 6 (Daxx), allowing Ets-1 to modulate the expression of the pro-angiogenic factors VEGFR-1, VEGFR-2, and epidermal growth factor-like domain 7 (EGFL-7; Murakami et al., 2006; Thakker et al., 2018). Another mechanism by which LANA enhances the expression of the transcription factors Ets-1 and Ets-2 is repression of cellular miRNAs miR-221 and miR-222 (Wu et al., 2011). This process contributes to the expression of pro-angiogenic genes.

Moreover, LANA increases the expression and secretion of Ang-2 and the extracellular matrix metalloproteinase inducer (EMMPRIN; also known as CD147), which promotes invasiveness by activating signaling pathways such as PI3K/Akt and MAPK/ERK (Sadagopan et al., 2009; Qin et al., 2010; Dai et al., 2012). In KSHV-infected endothelial cells, EMMPRIN also induces the metalloproteinases ADAMTS-1 (a disintegrin and metalloproteinase with thrombospondin motifs) and ADAMTS-9, and the pro-angiogenic protein heme-oxygenase (HO) 1 (Dai et al., 2016a,b). To conclude, LANA plays at least two essential roles in KSHV-infected cells: ensuring the maintenance of latency and promoting viral tumorigenesis in part by inducing angiogenesis.

### vFLIP and vCyclin

ORF71 and ORF72 are expressed together as they are located in a bicistronic mRNA and code for the viral FLICE (FADD-like interferon converting enzyme)-like inhibitory protein (vFLIP) and the cyclin D homolog, vCyclin, respectively (Dittmer et al., 1998). Both genes are expressed during latency, and their proteins modulate pro-survival and cell proliferation pathways. Introduction of vFLIP in endothelial cells modulates the expression of a number of genes that are essential for blood vessel development (Punj et al., 2009). Importantly, vFLIP can directly interact with IκB kinase (IKK) α, IKKβ, and IKKγ/ NF-κB essential modulator (NEMO) complexes, promoting IκBα destruction and thus NF-κB activation (Field et al., 2003; Guasparri et al., 2004). Additionally, it was recently demonstrated that vFLIP uses an alternative pathway to induce the activation of NF-κB by promoting the degradation of the histone deacetylase complex components SAP-18 and histone deacetylase (HDAC) 1, which target p65 for deacetylation (Ding et al., 2019). Importantly, activation of NF-κB is essential for vFLIP-induced endothelial cell migration, invasion, and angiogenesis (Ding et al., 2019). In an NF-κB-dependent manner, and in coordination with LANA, vFLIP increases expression of the epigenetic modifier EZH2, which in turn modulates the pro-angiogenic factor Ephrin-B2 (He et al., 2012).

Furthermore, in endothelial cells, vFLIP plays an essential role in KSHV-induced expression and secretion of COX-2 and PGE2 (Sharma-Walia et al., 2006, 2010; George Paul et al., 2010). This induction of COX-2 not only requires NF-κB but is also dependent on PI3K/Akt and p38/MAPK (Sharma-Walia et al., 2012). Finally, both vFLIP and vCyclin can induce expression of the host microRNA cluster miR-17/92, which targets Smad-2 and perturbs TGF-β signaling (Choi H. S. et al., 2015). This process is hypothesized to modulate viral-induced angiogenesis as, paradoxically, TGF-β has both pro- and anti-angiogenic functions (Principe et al., 2014). Although not much is known about the role of vCyclin in angiogenesis, these studies have demonstrated that mostly through the activation of NF-κB, vFLIP is important for KSHV-induced angiogenesis.

### Kaposins

The ORF K14 encodes Kaposin A, while Kaposins B and C transcription begins upstream of ORF K14. Kaposins A and C have not been thoroughly studied, although it has been shown that Kaposin A has oncogenic potential as it can induce focus formation in Rat-3 and NIH3T3 cells and form tumors in nude mice (Kliche et al., 2001; Chen X. et al., 2009). Additionally, the expression of Kaposin B results in the activation of pro-inflammatory and pro-angiogenic signaling involving p38/MK-2 and STAT-3 (McCormick and Ganem, 2005; King, 2013). Importantly, activation of p38/MK-2 promotes the accumulation of AU-rich element-containing mRNAs, including prospero homeobox protein 1 (PROX-1; Yoo et al., 2010). Consequently, this process allows for the stabilization of PROX-1 mRNA, which contributes to KSHV-induced lymphatic reprogramming of blood endothelial cells (Hong et al., 2004). Another mechanism by which Kaposin B modulates angiogenesis involves inactivation of the host miRNAs miR-221 and miR-222 in a c-Myc-dependent manner (Chang et al., 2016). As mentioned above, these two miRNAs target the transcription factors Ets-1 and Ets-2. To conclude, these studies highlight the roles of the Kaposins in mediating viral-induced pathogenesis and possibly contributing to tumor angiogenesis.

### KSHV MicroRNAs

Kaposi’s sarcoma-associated herpesvirus encodes 12 precursor microRNAs that are processed into at least 25 mature miRNAs that target viral and host mRNAs to maintain latency while also promoting tumorigenesis (Cai et al., 2005; Pfeffer et al., 2005; Samols et al., 2005). These miRNAs are expressed during latency and are abundant in KSHV-associated malignancies (Marshall et al., 2007; O’Hara et al., 2009a,b). In order to promote angiogenesis, several miRNAs target cellular anti-angiogenic and anti-proliferative proteins such as THBS-1 (Samols et al., 2007; Gallaher et al., 2013). KS tumor tissues show low levels of THBS-1, and several miRNAs have been identified to target its transcript, resulting in a reduction in TGF-β activity (Taraboletti et al., 1999; Samols et al., 2007).

Moreover, the KSHV miRNA K12-6-5p targets the breakpoint cluster region, enhancing Rac-1 activity, and promoting tubule formation (Ramalingam et al., 2015). Rac-1 belongs to the Rho family of GTPases involved in angiogenesis and reorganization of the cytoskeleton. Additionally, miRNA K12-6-5p targets the metastasis suppressor CD82, allowing c-Met to be activated, and induces endothelial cell invasion and angiogenesis (Li et al., 2017). In addition to the viral proteins known to activate the PI3K/Akt pathway (Bhatt and Damania, 2012), KSHV miR-K12-3 targets G protein-coupled receptor kinase 2 (GRK-2), promoting the activation of CXCR-2/Akt (Li et al., 2016a). This increase in Akt activity enhances the invasiveness and angiogenic potential of endothelial cells (Li et al., 2016a). Furthermore, KSHV miRNAs modulate the activation of the JAK/STAT signaling pathway. For example, expression of miR-K12-6-3p promotes the degradation of SH3 domain-binding glutamic acid-rich protein (SH3BGR), relieving STAT-3 repression and allowing nuclear translocation and expression of pro-angiogenic genes such as MMP-13, VEGF-A, and VEGFR-2 (Li et al., 2016b). Together, these studies illustrate the mechanisms that a handful of viral miRNAs utilize to promote tumorigenesis.

### K1

The ORF K1 is located on the extreme left end of the KSHV genome (reviewed in Sousa-Squiavinato et al., 2015). It is an immunoreceptor tyrosine-based activation motif (ITAM)-containing transmembrane glycoprotein with transforming capacities, able to immortalize endothelial cells (Lee et al., 1998; Wang et al., 2006). Through its ITAM, K1 recruits SH2-containing proteins such as Syk and phosphoinositide phospholipase C (PLC) γ-1 and induces their constitutive activation (Figure 4; Lagunoff et al., 1999; Lee et al., 2005). Expression of K1 in endothelial cells leads to an increase in activation of the PI3K/Akt pathway, resulting in the secretion of VEGF and consequently angiogenic activity (Tomlinson and Damania, 2004; Wang et al., 2004). Additionally, K1 can synergize with the HIV protein Nef to induce the expression of cellular miR-718, which targets PTEN and thus promotes the activation of the PI3K/Akt pathway, inducing angiogenesis in both *in vitro* and *in vivo* models (Xue et al., 2014). Similarly, through the modulation of another cellular miRNA, miR-891a-5p, K1 synergizes with HIV-1 Tat to promote angiogenic processes by activating NF-κB signaling (Yao et al., 2015). Thus, K1 activation of several signaling pathways, chiefly PI3K/Akt, promotes the angiogenic activity of endothelial cells.

### K15

Located at the right end of the viral genome, ORF K15 encodes a transmembrane protein composed of eight alternatively spliced exons (Choi et al., 2000). K15 is a functional homolog to EBV’s LMP2A, and its expression modulates pro-angiogenic cytokines and chemokines such as IL-6, IL-8, and CCL-20 (Brinkmann et al., 2007; Bala et al., 2012). The cytoplasmic tail of K15 contains SH2- and SH3-binding motifs that recruit and constitutively activate PLCγ-1 (Glenn et al., 1999). Moreover, K15 activates signaling pathways involving Ras, JNK, NF-κB and the transcription factors AP-1 and nuclear factor of activated T-cells (NFAT) (Figure 4; Brinkmann et al., 2003; Cho et al., 2008). Recruitment of PLCγ-1 and activation of NFAT are essential for K15 to induce pro-angiogenic phenotypes such as endothelial cell tubule formation (Bala et al., 2012; Gramolelli et al., 2015). This angiogenic process involves the expression of regulator of calcineurin (RCAN) 1, which is induced explicitly by K15 via activation of calcineurin (Bala et al., 2012). In sum, several studies have suggested that most of the pro-angiogenic signaling induced by K15 is mediated through the activation of NFAT.

### Viral Interleukin-6

The ORF K2 encodes viral interleukin-6 (vIL-6), and its expression can be detected at low levels during latency but increases upon lytic replication. As the name indicates, vIL-6 is the viral homolog of human IL-6, and they share some properties such as the ability to induce activation of the JAK/STAT signaling pathway (Moore et al., 1996; Molden et al., 1997; Neipel et al., 1997; Nicholas et al., 1997; Chen D. et al., 2009). However, in contrast to hIL-6, which requires both parts of the IL-6 receptor (IL-6R; gp80 and gp130), vIL-6 can induce signaling through gp130 in the absence of IL-6Rα (gp80). This allows the viral protein to constitutively activate the pathway promoting cell proliferation, migration, and angiogenesis (Aoki et al., 1999; Chen D. et al., 2009; Giffin et al., 2014, 2015; Wu et al., 2014). The involvement of vIL-6 in mediating angiogenesis was demonstrated when vIL-6-expressing NIH3T3 cells formed highly vascularized tumors in nude mice (Aoki et al., 1999). vIL-6 is an important player in the pathogenesis of Multicentric Castleman’s disease (MCD), as the viral cytokine can be detected in the serum of patients, induce MCD-like disease in mice, and promote secretion of hIL-6, a significant contributor to the disease (Mori et al., 2000; Aoki et al., 2001; Suthaus et al., 2012).

vIL-6 is sufficient, but not necessary, for KSHV-induced differentiation of blood to lymphatic endothelial cells (Morris et al., 2012). Importantly, the induction of the lymphatic markers PROX-1, VEGFR-3, and podoplanin (PDPN), expressed in KS tumor cells, requires the activation of both JAK/STAT and PI3K/Akt pathways (Morris et al., 2012). Moreover, in addition to the KSHV proteins mentioned above, vIL-6 also represses TGF-β2 signaling to levels seen with KSHV infection (DiMaio et al., 2014). The suppression of TGF-β2 is essential for viral-induced tubule formation (DiMaio et al., 2014). Similar to K1, vIL-6 has been found to synergize with HIV-1 proteins Tat and Nef to promote angiogenesis through activation of the PI3K/Akt pathway (Zhou et al., 2013; Zhu et al., 2014). vIL-6 is also involved in promoting endothelial cell migration, and possible angiogenesis, by activating proteins such as carcinoembryonic antigen-related adhesion molecule (CECAM) 1, hypoxia-upregulated protein (HYOU) 1, DNA methyltransferase (DNMT) 1, and CCL-2 (Giffin et al., 2014, 2015; Wu et al., 2014). In conclusion, a multitude of studies using both *in vitro* and *in vivo* models have demonstrated the highly pro-angiogenic functions of vIL-6 and how this viral protein might contribute to KSHV-associated malignancies.

### Viral G-Protein Coupled Receptor

ORF74 encodes the early lytic protein viral G-protein coupled receptor (vGPCR), the viral homolog to cellular CXCR-1 (Cesarman et al., 1996; Arvanitakis et al., 1997). Activation of the IL-8 receptor CXCR-1 is widely known to be pro-angiogenic in endothelial cells (Figure 2; Li et al., 2003). vGPCR activates major signaling pathways including p38, JNK, PI3K/Akt, MEK/ERK, JAK/STAT, and Wnt/β-cat, and the transcription factors AP-1, CREB, NFAT and NF-κB (Figure 4; Bais et al., 1998; Sodhi et al., 2000; Cannon et al., 2003; Burger et al., 2005; Angelova et al., 2014; Wong and Damania, 2017). Activation of these signaling pathways leads to the vGPCR-mediated secretion of pro-angiogenic factors such as IL-6, IL-8, GRO-α, CCL-3, CCL-4, stromal cell-derived factor 1 (SDF-1β), and VEGF (Montaner et al., 2004). In contrast to VEGF, whose expression is mainly dependent on HIF-1α, secretion of the other cytokines is dependent on the GTPase Rac-1 (Sodhi et al., 2000; Schwarz and Murphy, 2001; Montaner et al., 2004). In addition to the above-mentioned signaling pathways, the Hippo pathway has recently been highlighted as an important contributor to angiogenesis (Kim et al., 2017; Boopathy and Hong, 2019). vGPCR activates the downstream mediators of the Hippo pathway, Yes-associated proteins (YAP)/Tafazzin (TAZ), which in turn may facilitate the angiogenic process by mediating VEGFR signaling and expression of angiopoietin-2 (Choi H. J. et al., 2015; Liu et al., 2015).

Viral G-protein coupled receptor immortalizes HUVECs and induces the expression of VEGFR-2, which is vital for the survival of these cells (Montaner et al., 2001; Bais et al., 2003). Furthermore, vGPCR transforms NIH3T3 cells and promotes the formation of highly vascularized tumors in nude mice (Bais et al., 1998). Secreted factors from vGPCR-expressing cells, especially VEGF, induce tubule formation in endothelial cells, suggesting the involvement of paracrine signaling in promoting angiogenesis (Bais et al., 2003). Finally, conditioned media from vGPCR-expressing cells induce the expression in HUVECs of pyruvate kinase 2 (PKM-2), which acts as a coactivator of HIF-1 to promote aerobic glycolysis and tubule formation (Ma et al., 2015).

As a potent inducer of angiogenic processes, vGPCR modulates the expression of other pro-angiogenic proteins such as Ang-2, which is necessary for the survival of KSHV-infected endothelial cells, and angiopoietin-like (ANGPTL) 4, which promotes angiogenesis and vascular permeability in endothelial cells (Sadagopan et al., 2009; Ma et al., 2010; Hu et al., 2011). Additionally, in coordination with vIL-6 and dependent on MEK/ERK signaling, vGPCR induces the expression of angiopoietin-2 in lymphatic endothelial cells (Vart et al., 2007). Furthermore, through activation of Src, vGPCR induces membrane type (MT) 1-MMPs and represses TIMP-2, resulting in an increase of MMP-2 and arterial endothelial-cell tubule formation (Shan et al., 2007). Taken together, these studies demonstrate that vGPCR is a potent inducer of pro-angiogenic processes, and thus, it is expected to contribute significantly to KSHV-associated malignancies.

### Viral Interferon Regulatory Factors

Kaposi’s sarcoma-associated herpesvirus encodes four viral homologs to cellular interferon regulatory factors (IRFs) (reviewed in Jacobs and Damania, 2011). They play an essential role in inhibiting their cellular counterparts’ innate function. Although it is likely that all four vIRFs contribute to the pathogenesis of KSHV, especially by modulating Notch and TGF-β/Smad signaling, only vIRF3 has been identified to have a role in promoting angiogenesis (Jacobs and Damania, 2011). vIRF-3, also known as LANA-2, is a latently expressed protein that interacts with HIF-1α, promoting its stabilization and pro-angiogenic signaling including the expression of VEGF (Figure 4; Rivas et al., 2001; Shin et al., 2008). Furthermore, vIRF-3 can also interact with HDAC-5, sequestering it in the nucleus, and inducing expression of the lymphatic markers PDPN and PROX-1 (Lee et al., 2018). These markers, which are also induced in KSHV-infected endothelial cells, contribute to the spindle-shape form characteristic of KS (Lee et al., 2018). Finally, vIRF-3 is necessary for KSHV-induced lymphatic endothelial cell tubule formation and sprouting (Lee et al., 2018). Thus, in addition, to playing a role in repressing host antiviral response, vIRFs may contribute to KSHV-induced angiogenesis.

### Viral CC Chemokine Ligands

Kaposi’s sarcoma-associated herpesvirus encodes three CC chemokine ligands (CCLs), vCCL-1 (ORF K6), vCCL-2 (ORF K4), and vCCL-3 (ORF K4.1; Nicholas et al., 1997). These were previously known as viral macrophage inhibitory proteins (vMIPs). These viral proteins can interact with cellular CC-chemokine receptors and inhibit their signaling. All three vCCLs have been found to induce pro-angiogenic phenotypes in both *in vitro* and *in vivo* models (Boshoff et al., 1997; Stine et al., 2000; Cherqui et al., 2007). In particular, vCCL2 has been identified as capable of binding to multiple chemokine receptors, thus able to act on several cell types (Szpakowska and Chevigne, 2016). Therefore, vCCLs may play an essential role in the tumor microenvironment not only by acting on virus-infected cells but also by recruiting immune cells.

### Viral Protein Kinase

The viral protein kinase (vPK) encoded by ORF36 is one of two kinases expressed by KSHV (Park et al., 2000). The other kinase is the homolog of cellular thymidine kinase encoded by ORF21. The expression of vPK is detected mostly during lytic replication, although it can also be detected in the absence of full lytic replication. This expression during latency is hypothesized to occur during hypoxic conditions as HIF proteins upregulate vPK expression via HIF response elements located at the promoter of the ORF34–37 cluster (Haque et al., 2006). Notably, vPK activates the JNK pathway by phosphorylating mitogen-activated protein kinase kinase (MKK)-4 and MKK-7 (Hamza et al., 2004).

Furthermore, vPK mimics the activity of the cellular protein S6 kinase β1 (P70S6K1), promoting protein synthesis by activating ribosomal protein S6 and eukaryotic initiation factor 4B (EIF4B; Bhatt et al., 2016). Both S6 and EIF4B are downstream components of the PI3K/Akt pathway that promote translation of HIF-1α, enhancing angiogenesis (Karar and Maity, 2011). Importantly, expression of vPK augments anchorage-independent growth and promotes endothelial cell tubule formation (Bhatt et al., 2016). In addition to its oncogenicity *in vitro*, vPK expressed in transgenic mice generates a hyperproliferation of B-cells and a higher incidence of B-cell non-Hodgkin’s lymphomas (Anders et al., 2018). Together, these studies demonstrate a tumorigenic role for this viral kinase and its possible involvement in promoting angiogenesis.

## *In Vivo* Models to Study EBV- and KSHV-Induced Angiogenesis

The lack of tropism for murine cells by both EBV and KSHV has required the development of methods to study viral-induced malignancies *in vivo*. Most *in vivo* studies rely on the engraftment of tumor or viral protein-expressing cells into mice, or transgenic mice expressing the viral proteins. Another approach to study the viruses *in vivo* involves the humanization of the mice, which makes the animal permissive for viral infection. These studies have proven to be useful for the characterization of viral proteins and RNAs, especially in the context of lymphomagenesis. Several recent reviews published elsewhere had discussed these *in vivo* models in detail (Dittmer et al., 2015; Fujiwara et al., 2015; Ahmed and Baiocchi, 2016; Purushothaman et al., 2016; Munz, 2017; Fujiwara, 2018; Bravo Cruz and Damania, 2019).

Transgenic mice expressing viral proteins such as KSHV vGPCR demonstrates the involvement of this protein in inducing highly angiogenic KS-like lesions (Holst et al., 2001; Guo et al., 2003). Furthermore, conditional transgenic mice have also been created, allowing for the doxycycline-inducible expression of viral genes (Jensen et al., 2005; Grisotto et al., 2006). To restrict the expression to specific cell types, such as endothelial cells, the Tie2-tva transgenic mice was developed. This mouse model expresses the avian leukosis virus (ALV) receptor, Tva, under a Tie2 promoter restricting the expression of the receptor to endothelial cells (Montaner et al., 2003). These cells are permissive to infection with ALV-derived retroviruses expressing a viral gene of interest. Similar to the previous model, expression of vGPCR in these cells also led to the formation of highly angiogenic KS-like lesions (Montaner et al., 2003; Sodhi et al., 2004, 2006).

The models to study EBV-induced angiogenesis *in vivo* have not been developed to the same extent as with KSHV. Most of these models consist of the injection of EBV-positive NPC cells or EBV-transformed LCLs into immunocompromised mice (Hong et al., 2005b; Smith et al., 2011; Yang et al., 2015; Ma et al., 2018; Ye et al., 2018). Although these models have not been extensively used for angiogenic studies, they have provided vital knowledge with regards to pro-tumorigenic viral factors.

Two cell lines used for *in vitro* and also *in vivo* studies are the telomerase-immortalized vein endothelial (TIVE) cells or the BAC36-transfected murine cells (mECK36), which are suitable models due to their ability to grow and resemble KS *in vivo* (An et al., 2006; Mutlu et al., 2007). These models allow for the characterization of essential cellular and viral factors that contribute to tumorigenesis and also for the testing of possible therapeutic compounds. For studying PEL, several cell lines, notably BCBL-1, injected into immunocompromised mice give rise to tumors. Even though this model induces the formation of a solid tumor, cells produce an accumulation of ascites resembling PEL and allowing for the studies of anti-tumorigenic compounds. Furthermore, patient-derived xenografts were recently established to study NPC (Lin et al., 2018). Altogether, these technological advances have provided suitable tools to study viral-induced malignancies in animals.

The chicken chorioallantoic membrane (CAM) assay has been widely used to study angiogenesis *in vivo*. This assay is simplistic due to the nature of the highly vascularized extraembryonic membrane following the fertilization of the egg (Nowak-Sliwinska et al., 2014). Cells, proteins, or compounds can be incorporated into the membrane allowing for the assessment of the effect of these factors in mediating angiogenesis. The whole embryo can be removed from the eggshell and placed on a petri dish to facilitate the imaging process. In fact, this method has been used by several groups to demonstrate KSHV-induced angiogenesis (Stine et al., 2000; Zhou et al., 2013; Zhu et al., 2014). Thus, the CAM assay provides a cost-effective approach to study angiogenesis in an *in vivo* context.

## Targeting Angiogenesis in EBV- and KSHV-Associated Malignancies

Research into EBV- and KSHV-induced angiogenesis has suggested an essential role for this process in viral tumorigenesis. As discussed above, virus infection or expression of viral proteins and non-coding RNAs have already been identified as contributing to this process. These studies have paved the way for several clinical trials of compounds with anti-angiogenic activity to treat viral malignancies, particularly KS and NPC.

Since the mid-2000s, several inhibitors with anti-angiogenic activity, including chemical and biological agents, have been clinically approved for different types of cancers (Ribeiro et al., 2018). These include the VEGF inhibitor bevacizumab (Avastin), VEGFR-2 antagonist ramucirumab (Cyramza) and several receptor tyrosine kinase (RTK) inhibitors that target VEGFRs, c-Met, and PDGFR, such as sunitinib (Sutent) and cabozantinib (Cabometyx). Also, the immunomodulatory compounds thalidomide (Immunoprin) and lenalidomide (Revlimid) used to treat multiple myeloma show anti-angiogenic activity by targeting angiogenic inducers such as IL-6, NF-κB, COX-2, and VEGF (Ribeiro et al., 2018).

Several of these inhibitors have been tested or are currently undergoing clinical trials against EBV- and KSHV-associated malignancies. For example, the switch of immunosuppressant from cyclosporin A to the mTOR inhibitor rapamycin (Sirolimus) elicited KS regression in transplant patients, and it is now the first line of treatment for transplant KS (Stallone et al., 2005). The PI3K/Akt/mTOR pathway has proven to be vital for KS and PEL, and several inhibitors have proven to be efficacious in pre-clinical studies (Sin et al., 2007; Bhatt et al., 2010; Roy et al., 2013). Furthermore, Uldrick et al. (2012) reported that out of 16 KS patients treated with bevacizumab, 31% showed complete or partial response. However, in another small group of patients, although well tolerated, intralesional administration of bevacizumab had no significant effect against upper respiratory tract KS (Ablanedo-Terrazas et al., 2015). Thus, targeting VEGF with bevacizumab has a moderate effect against KS.

Additionally, targeting the receptor tyrosine kinases, c-kit, and PDGFR, with the inhibitor imatinib (Gleevec), provided a long-term benefit to a third of patients suffering from the most aggressive KS subtype, AIDS-KS (Koon et al., 2014). Moreover, 16 of 22 KS patients that were treated with pomalidomide responded well and rapidly to the treatment (Polizzotto et al., 2016). Additionally, several of these immunomodulatory compounds that show anti-tumorigenic and anti-angiogenic activity are currently being studied for treatment against KSHV- and EBV-associated lymphomas (NCT02911142). Furthermore, the anti-IL-6 antibody siltuximab (Sylvant) has been FDA approved for idiopathic MCD, and the anti-IL-6R (gp80) antibody tocilizumab (Actemra) is currently been tested in KSHV-associated MCD patients (NCT01441063; van Rhee et al., 2014).

In NPC patients, the addition of bevacizumab to standard chemoradiation proved to be safe and suggested that the treatment might delay the progression of the disease (Lee et al., 2012). Furthermore, the combination of the recombinant endostatin (Endostar), which inhibits VEGF, with gemcitabine and cisplatin increased the overall survival of metastatic-NPC patients (Jin et al., 2018). In contrast, the use of the EGFR inhibitor erlotinib (Tarceva) as maintenance following gemcitabine plus platinum-based chemotherapy was ineffective in recurrent or metastatic NPC patients (You et al., 2012). Thus, the addition of these anti-angiogenic compounds in combination with standard chemotherapy may become a standard treatment for NPC patients.

Importantly, apart from the anti-VEGF therapies, most of these therapeutics were not developed as anti-angiogenic. Nevertheless, their effect in reducing angiogenesis is predicted to be a product of inhibiting pathways known to contribute to angiogenic processes. Thus, these clinical studies have highlighted the potential benefit of several different therapeutic approaches that may contribute to reducing pro-angiogenic mechanisms. Consequently, more studies are currently underway and as new anti-angiogenic therapeutic approaches are developed, we can expect that patients suffering from EBV- and KSHV-associated malignancies will eventually benefit from these treatments.

## Concluding Remarks and Future Perspectives

Angiogenesis is one of the hallmarks of cancer, and the ability of a tumor to hijack this process for its benefit facilitates growth and enhances its ability to metastasize (Hanahan and Weinberg, 2011). Viral cancers are not an exception (Mesri et al., 2014). Two of the seven known human oncoviruses, EBV and KSHV, belong to the *Gammaherpesvirinae* subfamily. Both viruses are linked to several malignancies, including both solid and liquid tumors. Importantly, both viruses are known to have pro-angiogenic activity that enhances their tumorigenesis.

Most of the research into EBV- and KSHV-induced angiogenesis has been accomplished just in the last two decades. However, several viral factors, especially in KSHV, have already been identified as playing a direct and substantial role in modulating pro-angiogenic processes, leading to clinical trials using anti-angiogenic inhibitors. The fact that the induction of angiogenesis is not restricted to a single viral factor suggests the importance of this process in mediating viral pathogenesis. Specifically, both latent and lytic proteins are involved in this process, supporting the hypothesis that paracrine signaling is vital for these viral-induced malignancies. In KSHV-associated malignancies, the current knowledge points to lytic proteins such as vGPCR and vIL-6 to be amongst the most potent pro-angiogenic inducers. However, in the context of EBV tumorigenesis, the latent protein LMP1, appears to be the major pro-angiogenic factor. Interestingly, EBV encodes a constitutively active GPCR known as BILF1 that has not been exhaustively studied, although it is known that it can induce tumors in nude mice (Paulsen et al., 2005; Lyngaa et al., 2010). It would be of significant interest to determine whether BILF1 contributes to EBV-induced angiogenesis.

The emerging field of non-coding RNAs, which includes both cellular and viral miRNAs, long non-coding (lnc) RNAs, and circular (circ) RNAs, may be significant contributors to angiogenesis (Khorshidi et al., 2016). As discussed above, EBV and KSHV express miRNAs that have been demonstrated to be involved in mediating angiogenesis. Recently, a few cellular lncRNAs have been identified to be regulated during hypoxia and contributing to tumor angiogenesis (Yu and Wang, 2018). Additionally, circRNAs may also be important players in regulating this pathological process (Su et al., 2019). As both viruses, EBV and KSHV, express these types of non-coding RNAs, it would be of significant interest to determine whether, in addition to miRNAs, the other non-coding RNAs contribute to viral-induced angiogenesis (Tagawa et al., 2018; Toptan et al., 2018; Ungerleider et al., 2018, 2019). Importantly, the development of animal models, including mouse and zebrafish, are promising methods for the *in vivo* characterization of these non-coding RNAs (Feyder and Goff, 2016).

Furthermore, new techniques to study angiogenesis *in vitro* are currently being developed. One such tool consists of the “organ-on-chip” technology that allows for the study of vascularized microtumors (VMT) using real-time fluorescence microscopy (Sobrino et al., 2016). This *in vitro* 3D model may become useful not only for studying the vasculature but also for the screening of compounds with anti-angiogenic potential against viral-induced malignancies. These assays, in coordination with the development of new therapeutics targeting cellular pathways involved in angiogenesis, may provide a positive outlook for patients suffering from EBV- or KSHV-associated malignancies. This is vital given that most clinical trials with anti-angiogenic compounds have only been partially effective, raising the question on why cancers that appear to be highly dependent on angiogenesis such as KS can still be, to some extent, resistant to this type of therapy. A major hypothesis arises from the fact that viral factors induce a multitude of oncogenic processes besides angiogenesis. These include the induction of cell proliferation and inhibition of apoptosis. Thus, targeting multiple cellular pathways or combining inhibitors against both cellular and viral proteins is hypothesized to reduce the development of resistance, which is not uncommon with single-agent treatments. We predict that in the near future, research into EBV- and KSHV-induced angiogenesis will further delineate the mechanisms by which cellular and viral factors cooperate to modulate the pathological process, and thus provide additional treatment targets to improve the patient’s quality of life.

## Author Contributions

Both authors wrote and edited this review.

## Conflict of Interest Statement

The authors declare that the research was conducted in the absence of any commercial or financial relationships that could be construed as a potential conflict of interest.
